# The influence of extra-articular changes on hip function in young adults with a history of Perthes disease

**DOI:** 10.1177/11207000261420153

**Published:** 2026-03-05

**Authors:** Johan Olav Brevik, Kristine Risum, Armend Fejzulai, Terje Terjesen, Ola Wiig, Stefan Huhnstock

**Affiliations:** 1 Section of Orthopaedic Rehabilitation, Division of Orthopaedic Surgery, Oslo University Hospital, Oslo, Norway; 2 Department of Rehabilitation Science and Health Technology, Faculty of Health Sciences, Oslo Metropolitan University, Oslo, Norway; 3 Department of Orthopaedics and Traumatology, Oestfold Hospital Trust, Østfold, Norway; 4 University of Oslo Institute for Clinical Medicine, Oslo, Norway; 5 Section of Children's Orthopaedics and Reconstructive Surgery, Division of Orthopaedic Surgery, Oslo University Hospital, Oslo, Norway

**Keywords:** Articulo-trochanteric distance, femoral offset, hip function, Perthes disease, Trendelenburg sign

## Abstract

**Background:**

Residual deformities after Perthes disease, such as high-rising greater trochanter (HGT) and reduced femoral offset (FO), may compromise abductor function and influence long-term hip function.

**Purpose:**

To determine the prevalence of HGT and abnormal FO in young adults with healed unilateral Perthes disease and evaluate their associations with the Stulberg classification and hip function.

**Methods:**

In this cross-sectional follow-up study, 180 individuals (mean age 28.2 years, 72% male) previously diagnosed with unilateral Perthes disease were examined with radiographs and clinical tests. Articulo-trochanteric distance (ATD) and FO were measured on calibrated pelvic radiographs. Femoral head shape was classified using a modified 3-group Stulberg classification. Hip function was evaluated using the Trendelenburg test, passive hip abduction range of motion, and the Copenhagen Hip and Groin Outcome Score (HAGOS).

**Results:**

ATD was significantly lower in Perthes hips compared to contralateral hips (7.7 mm [SD 9.6] vs. 20.7 mm [SD 6.2], *p* < 0.001). HGT was present in 70 individuals (39%), and a positive Trendelenburg test was observed in 12 Perthes hips (7%). Risk factors for a positive test included hip pain, aspherical femoral head, and surgical treatment. Mean FO was lower in Perthes hips (34.2 mm vs. 39.7 mm, *p* < 0.001), as was hip abduction (25.5° vs. 29.7°). ATD and FO were significantly associated with the modified Stulberg classification, particularly between Stulberg 1 and Stulberg 3 hips (*p* < 0.001). No significant associations were found between ATD, FO, and hip function parameters, nor between HGT and HAGOS scores in spherical hips.

**Conclusions:**

Reduced ATD and FO were prevalent in young adults with previous PD and were associated with the modified Stulberg classification but not with hip function.

**Clinical trials registration:**

ClinicalTrials.gov (NCT03995960).

## Introduction

Perthes disease (PD) is a hip disorder affecting children aged 2–12 years.^1^ It is characterised by a partial or complete loss of blood supply to the epiphysis of the femoral head. Despite potential growth alterations of the femoral neck, the greater trochanter (GT) develops unaffected, causing a disproportionate overgrowth.^2^ This growth imbalance may lead to a high-rising greater trochanter (HGT) and a reduced FO, which can be deteriorated by surgical treatment such as proximal varus femoral osteotomy (FVO).^3^

While deformities occur during the active phases of the disease, their clinical consequences may persist into adulthood.^3^ HGT can cause shortening of the gluteus medius and minimus muscles, leading to abductor muscle insufficiency, often presented by a Trendelenburg sign or gait.^3^ Earlier studies have indicated a link between reduced articulo-trochanteric distance (ATD) and a positive Trendelenburg sign.^2,4,5^ Edgren^2^ noted that the Trendelenburg sign often became positive when the ATD was less than 0, while Leitch et al.^4^ emphasised that an ATD < 5 mm resulted in a positive Trendelenburg sign. Another radiographic parameter that has been linked to abductor muscle insufficiency is FO,^6^ which has previously not been evaluated in PD.

Knowledge of the prevalence of proximal femoral deformities after PD and their association with hip strength and mobility, is scarce. Therefore, this study aims to: (1) establish the prevalence of HGT and abnormal FO in a large PD population of young adults; (2) investigate the clinical consequences of such radiographic changes.

## Patients and methods

**Figure 1.**
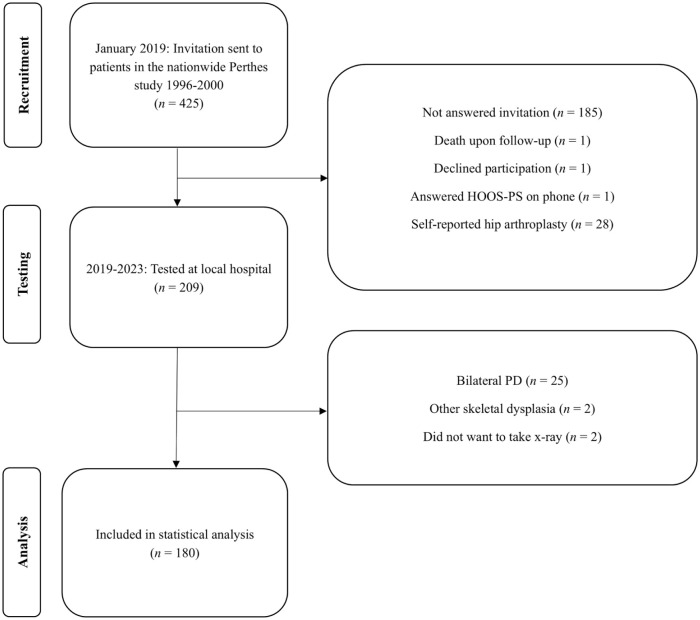
Flowchart of participants included in the study.

This cross-sectional study investigated the association between proximal femoral deformities and hip function in young adults with a history of Perthes disease. The cohort originated from a nationwide Norwegian multicentre study that prospectively included 425 patients between 1996 and 2000.^1^ Participants were treated either surgically with proximal femoral varus osteotomy (FVO) or non-surgically using Scottish Rite orthosis or physiotherapy. All patients were followed for 5 years. In 2018, they were invited to a follow-up study. Exclusion criteria were bilateral PD, total hip arthroplasty (THA), death during the follow-up period, and inability to provide consent (Figure 1). 180 participants were eligible for inclusion. They were examined between January 2019 and April 2023. 45 patients (25%) were treated surgically and 135 (75%) were non-surgically treated. Both the PD hip and the contralateral normal hip were examined.

### Radiographic measurement

**Figure 2.**
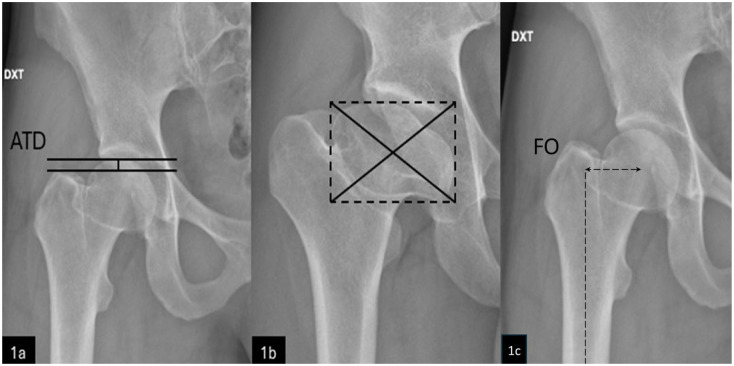
Radiographic assessment of 2 Perthes hips: (a) ATD: Distance between the 2 horizontal lines (solid). (b) Definition of centre of rotation: a rectangle is drawn outlining the medial, lateral, upper, and lower border of the femoral head (dotted lines), and diagonals (solid lines) mark the centre. (c) Femoral offset (FO): Distance from the centre of rotation of the femoral head to a line bisecting the long axis of the femur (dotted lines).

Standard anteroposterior (AP) pelvic and frog-leg lateral radiographs with calibration markers were obtained. Measurements were made using the hospital’s Sectra picture-archiving system (Sectra Workstation, Version 25.2, Sectra AB, Sweden, 2024). Radiographic analysis included both PD and non-affected hips, performed by 2 authors (SH and AF) after standardised calibration. ATD was measured as described by Edgren^2^ as the vertical distance from the greater trochanter tip to the femoral head’s proximal tangent (Figure 2). HGT was defined as ATD < 5 mm.^4^

Accurate centre-of-rotation identification is crucial for measuring FO. In severely deformed femoral heads, the centre was estimated by drawing a rectangle around the femoral head’s borders and intersecting diagonals (Figure 2). FO was defined as the horizontal distance from the centre of rotation to a line bisecting the femoral shaft’s longitudinal axis,^6^ as shown in Figure 2. Normal FO was calculated as the means of anatomically normal hips, with abnormal FO defined as values >2 standard deviations (SDs) below this mean.

Femoral head deformity was classified by 2 of the authors (SH and AF), using the modified 3-group Stulberg classification.^7^ It divides hips into round, ovoid, or flat, depending on the shape of the femoral head.

### Functional assessment

**Figure 3.**
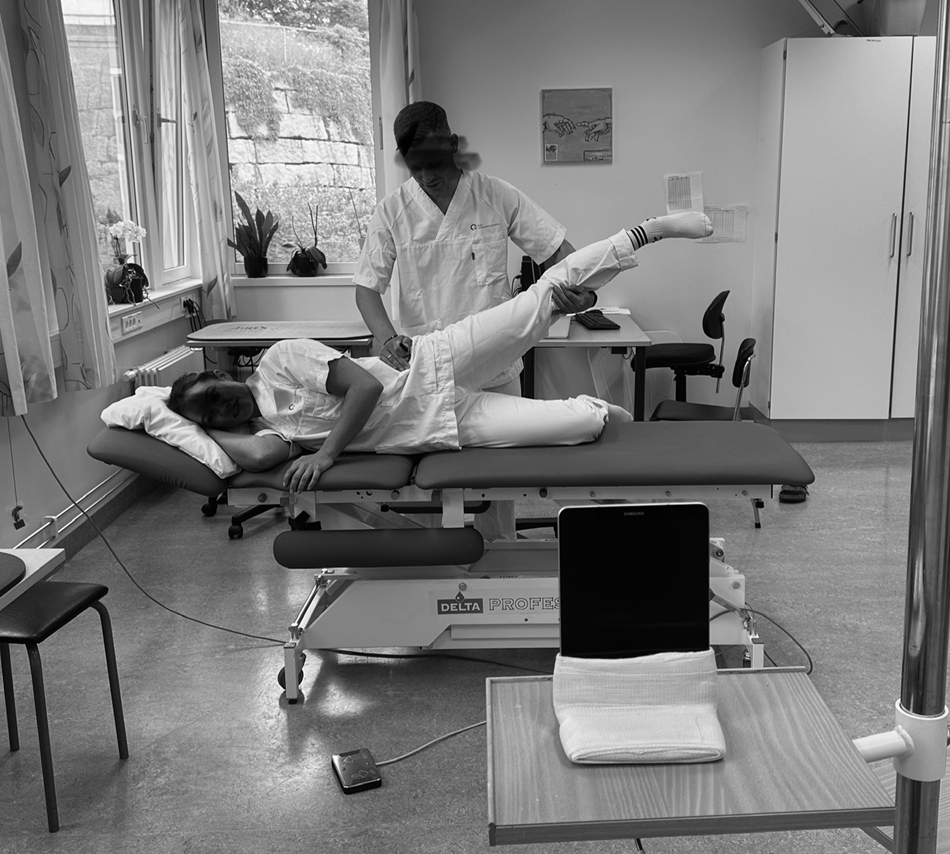
Illustration of hip abduction ROM measurement.

Functional assessments were performed by the same physiotherapist (JB) for consistency. Hip abduction range of motion was measured with the participant side-lying (Figure 3). The examiner stabilised the pelvis and passively abducted the hip. A remote-triggered tablet, 2 m away, captured an image of the entire lower limb –including the pelvis and foot – at maximum abduction without pelvic movement. Images were analysed using the Angulus app (Angles on Image/Video, Version 4.3.2, Android OS), which has demonstrated accuracy and precision comparable to traditional visual estimation and goniometry.^8^

Hip abductor strength was assessed with the Trendelenburg test, as described by Hardcastle and Nade.^9^ The examiner instructed the participant to lift 1 foot, flex the stance hip to 30°, and hold the position for 30 seconds without compensatory trunk lean. Both hips were tested, starting with the unaffected side. A positive test – indicating abductor insufficiency – was defined by a pelvic drop to the opposite side or lateral trunk lean.

### HAGOS questionnaire

The Copenhagen Hip and Groin Outcome Score (HAGOS) subdomains – Activities of Daily Living (ADL), Sport/Recreation, Physical Activity (PA), and Pain – were used to assess hip function.^10^ Scores range from 0 to 100, with higher scores indicating better function. To evaluate the impact of extraarticular changes on HAGOS scores, we included only the 92 PD patients with a round femoral head. For pain as a risk factor for a positive Trendelenburg test, all 180 patients were included.

### Statistical analysis

Normally distributed continuous data are presented as means with SD or ranges, and skewed data as medians with interquartile ranges (IQR). Normality was assessed using the Kolmogorov-Smirnov test and histogram inspection. Group comparisons for continuous variables were made with the paired samples *t*-test for normal data and the Mann-Whitney U-test for skewed data. 1-way analysis of variance (ANOVA) with post hoc testing was used for comparisons of more than 2 groups. Correlations were evaluated using Pearson’s for normal data and Spearman’s for skewed data. Categorical data are presented as frequencies and percentages. Univariable logistic regression was used to identify risk factors for a positive Trendelenburg test. All analyses were conducted using IBM SPSS Statistics version 29.0 (IBM Corp., Armonk, NY). A *p*-value < 0.05 was considered significant.

### Ethical considerations

Ethical approval was granted by the Regional Committee for Medical and Health Research Ethics, Norway (REK Southeast A 2018/1924-3, 25.09.2018), and the study registered at ClinicalTrials.gov (NCT03995960). All participants provided written consent.

## Results

A total of 180 patients were included in the study, 129 males (72%) and 51 females with a mean age of 28.3 (±2.5) years. The study participants showed no significant differences in age at diagnosis (*p* = 0.36), gender (*p* = 0.26), treatment type (*p* = 0.69), or Stulberg 3 group at the 5-year follow-up (*p* = 0.12) compared with those who did not participate in this study. The distribution of hips according to the 3-group Stulberg classification was round femoral head in 92 hips (51%), ovoid in 50 (28%), and flat femoral head in 38 hips (21%).

### Radiographic results

The mean ATD in Perthes hips was 7.7 mm (SD 9.6) and in normal contralateral hips 20.7 mm (SD 6.2; *p* < 0.001) (Table 1). 70 individuals (39%) with PD had HGT defined as ATD < 5 mm. Perthes hips had significantly lower FO compared to unaffected hips (mean 34.2 mm vs. 39.7 mm; *p* < 0.001). ATD and FO decreased significantly with increasing Stulberg groups (Table 1). Compared with non-surgically treated hips, surgically treated hips had lower ATD (4.3 vs. 8.5; *p* = 0.032) and higher FO (37.2 vs. 33.3; *p* = 0.009).

**Table 1.** Radiographic and clinical measurements in Perthes hips and unaffected hips, and the associations between the parameters and the modified Stulberg groups.

Variables	Normal hips (*n* = 180)	Mean - 2 SD	Perthes hips (*n* = 180)	Stulberg group			Significance level			
				I (*n* = 92)	II (*n* = 50)	III (*n* = 38)	p1	p2	p3	p4
ATD (mm)	20.7 (6.2)	8.3	7.7 (9.6)	12.4 (9.0)	4.7 (7.8)	0.4 (7.2)	0.001	<0.001	<0.001	0.045
FO (mm)	39.7 (6.6)	26.5	34.2 (8.1)	36.5 (7.3)	33.5 (7.7)	29.5 (8.8)	0.001	0.072	<0.001	0.047
Abduction (°)	29.7 (5.8)	18.1	25.5 (6.3)	27.1 (5.8)	24.7 (5.7)	22.5 (6.7)	0.001	0.055	<0.001	0.203

P1, comparison between all normal hips and Stulberg I hips; P2, comparison between Stulberg I and II hips; P3, comparison between Stulberg I and III hips; P4, comparison between Stulberg II and III hips; ATD, articulo-trochanteric distance; FO, femoral offset.

### Functional results

A positive Trendelenburg test was observed in 12 individuals with PD (7%) (Table 2). Positive Trendelenburg test was significantly associated with aspherical femoral head (Stulberg groups II and III), surgical treatment, and hip pain evaluated by HAGOS (Table 2).

**Table 2.** Risk factors for positive Trendelenburg test.

Variable	Group	*n*	Trendelenburg test		Significance level		
			Negative (*n* = 168)	Positive (*n* = 12)	*p*	OR	95% CI
HGT	ATD ⩾5 mm	110	104	6 (6%)	0.42	0.62	0.19–2.00
	ATD <5 mm	70	64	6 (8%)			
FO	Normal	149	138	11 (7%)	0.41	2.40	0.30–19.23
	Abnormal	31	30	1 (3%)			
Treatment	Non-surgical	142	136	6 (4%)	0.02	4.26	1.28–14.05
	Surgical	38	32	6 (16%)			
Stulberg	I	92	90	2 (2%)	0.04	2.10	1.02–4.30
	II	50	44	6 (12%)			
	III	38	34	4 (10%)			
Gender	Male	129	121	8 (8%)	0.70	1.29	0.37–4.50
	Female	51	47	4 (6%)			
HAGOS pain; mean score (range)		179	93 (5-100)	65 (13-95)	<0.001	0.95	0.94-0.98

HGT, high-rising greater trochanter, ATD, articulo-trochanteric distance; FO, femoral offset; FO Normal >26.5 mm; p = univariable logistic regression, OR, odds ratio, CI, confidence interval.

The mean hip abduction was significantly lower in Perthes hips than in nonaffected hips (25.5° vs. 29.7°; P < 0.001). Hip abduction was significantly lower in Stulberg group III than in group I (*p* < 0.001). There were no significant associations between hip abduction and the radiographic measurements ATD and FO.

HAGOS was used for the 92 participants with a round femoral head (Table 3). The mean scores of the 4 subscales were high: ADL (100), Sports/Rec (94), PA (100) and Pain (92). There were no significant differences in HAGOS subscale scores and the presence or absence of HGT (Table 3).

**Table 3.** Comparison of HAGOS subscores between patients with and without HGT in patients with a round femoral head.

HAGOS domains	HGT (*n* = 17) Median (range)	Normal (*n* = 75) median (range)	*p*
HAGOS ADL (0–100)	100 (90–100)	100 (45–100)	0.21
HAGOS Sport/Rec (0–100)	94 (69–100)	94 (16–100)	0.27
HAGOS PA (0–100)	100 (38–100)	100 (38–100)	0.92
HAGOS Pain (0–100)	92 (60–100)	97 (35–100)	0.41

HAGOS, the Copenhagen hip and groin outcome score subscale; HGT, high-rising greater trochanter; ADL, activities of daily living; Sport/Rec, sport and recreation, PA, physical activity.

## Discussion

The study revealed a high prevalence of HGT and reduced FO in young adults with previous PD, significantly associated with an aspherical femoral head (Stulberg group II–III). No significant associations were found between radiographic findings and hip function evaluated by abductor range of motion and Trendelenburg test.

We found that 39% had HGT, somewhat higher than the 21–23% previously reported.^4,11,12^ However, the mean ATD of 8 mm is similar to that of Larson et al.,^13^ who also found a mean ATD of 8 mm. We observed significantly lower ATD and higher prevalence of HGT in surgically treated patients compared to non-surgical, consistent with previous studies.^12,14^ While HGT in the non-surgical group can be attributed to femoral neck growth inhibition alone, the HGT in the surgical group is a combined result of this factor and varus angulation.

Only 7% of PD hips in our study had a positive Trendelenburg test, lower than earlier reported prevalences (19–28%).^2,4,13^ However, in a study based on 3-dimensional gait analysis, Anable et al.^15^ found that 8% of young adults with previous PD had a dynamic pelvic drop >5.4°, which is in accordance with our results at the static Trendelenburg test. If the 28 patients who already had undergone THA and thus represented the worst hips had been examined before THA, our prevalence would be somewhat higher than 7%. Earlier reports have described the widely recognised role of the Trendelenburg test in assessing hip abductor muscle function.^2,4,13^ Leitch et al.^4^ found that 23% of individuals with HGT had a positive Trendelenburg test, while Edgren^2^ suggested that an ATD of ⩽5 mm almost always resulted in a positive test, contrasting with our finding of 9% of the hips with HGT. These discrepancies highlight possible limitations of the Trendelenburg test, such as pain and lack of cooperation.

We have found no previous study that evaluated possible risk factors for a positive Trendelenburg test. The strongest independent risk factor in this study was hip pain, which was not unexpected, considering the weakening effect pain would have on hip abductor strength. Aspherical femoral head (modified Stulberg groups II and III) was also a risk factor, which is consistent with poorer long-term prognosis in these hips.^16^ We also identified surgical treatment as a risk factor, probably caused by reduced ATD after the FVO procedure.

Perthes hips had significantly lower FO compared with nonaffected hips. This might lead to gluteal muscle insufficiency. There is a lack of studies investigating FO in PD. Carmona et al.,^17^ examining the upper femoral anatomy in 628 healthy hips of individuals in their 3rd and 4th decades of life, found an average FO of 39–40 mm, similar to the mean FO of 40 mm in the nonaffected hips in our study. We found no significant association between FO and the Trendelenburg test, whereas Asayama et al.^18^ reported a significant correlation between FO and objectively measured hip abductor strength in patients following total hip arthroplasty, with reduced FO associated with a positive Trendelenburg sign. It is possible that the difference in FO between PD hips and nonaffected hips was not severe enough to influence abductor strength evaluated by the Trendelenburg test.

We found a decrease in abduction range of motion in PD hips, and the decrease was most pronounced in Stulberg III hips. This is in accordance with previous reports, where a decrease in the range of abduction was associated with higher degrees of femoral head deformity.^19^

Almost all patients with a round femoral head reported high scores in the 4 HAGOS subscores, and there was no association between HGT and the HAGOS scores. This suggests that HGT did not seem to impair patient-reported functional outcomes. Our findings align with those of Ali et al.,^20^ who reported that a spherical femoral head was associated with a high functional outcome.

This study had some limitations. First, we used the Trendelenburg test to assess hip abductor strength and pelvic stability. Even though this is a widely used clinical test, validation studies have shown no significant association between decreased isometric measured hip abductor strength and a positive Trendelenburg test.^21^ If we had used motion capture or video analysis instead of visual estimation, more than 7% of hips would probably have exhibited reduced abduction strength. Additionally, the Trendelenburg test provides only a static, momentary snapshot of the hip abductor function. The patients tested in our study (3^rd^ decade of life) had time to adapt and functionally compensate for their anatomical shortcomings,^5^ which might explain that they tested negative in a 30 second test. However, Trendelenburg test does not reveal pelvic instability due to muscle fatigue induced by prolonged activity, which might be a relevant problem in this patient group. Second, the HAGOS questionnaire, which is validated for young to middle-aged adults and addresses both hip and groin issues, showed a significant ceiling effect in the HAGOS subscores, suggesting that the instrument might not be sensitive enough to detect small changes in PD patients. Third, we did not analyse HAGOS subscale scores in patients with aspherical femoral head, since we considered that a deformed femoral head might have an influence on patient-reported outcomes.^20^

There are also some strengths in the study. First, the number of participants was relatively large, probably the largest cohort so far of young adults with previous PD examined with radiographic and functional assessments. Second, all radiographs were measured in a standardised manner with calibrated radiographs. 2 observers classified the radiographs separately (Stulberg), with a third observer in cases of disagreement. Third, all functional tests were performed by the same specially trained physiotherapist, which probably helped to reduce the risk of systematic measurement errors. Finally, hip abduction ROM was measured with digital photography, which allowed measurements of ROM several times.

In conclusion, young adults previously diagnosed with PD showed a relatively high prevalence of proximal femoral deformities; however, the clinical impact was limited, with few (7%) exhibiting a positive Trendelenburg test.
